# Eravacycline use in immunocompromised patients: multicenter evaluation of timely versus late initiation on clinical outcomes

**DOI:** 10.1128/spectrum.00565-25

**Published:** 2025-08-26

**Authors:** Ashlan J. Kunz Coyne, Sara Alosaimy, Kristen Lucas, Taylor Morrisette, Kyle C. Molina, Alaina DeKerlegand, Melanie Rae Schrack, S. Lena Kang-Birken, Athena L. V. Hobbs, Jazmin Agee, Nicholson B. Perkins, Mark Biagi, Michael Pierce, James Truong, Justin Andrade, Jeannette Bouchard, Tristan Gore, Madeline A. King, Benjamin M. Pullinger, Kimberly C. Claeys, Shelbye Herbin, Reese Cosimi, Serina Tart, Michael P. Veve, Bruce M. Jones, Leonor M. Rojas, Amy K. Feehan, Jing J. Zhao, Paige Witucki, Michael J. Rybak

**Affiliations:** 1 Anti-Infective Research Laboratory, Department of Pharmacy Practice, Eugene Applebaum College of Pharmacy and Health Sciences, Wayne State University 2954, Detroit, Michigan, USA; 2 Department of Emergency Medicine, University of Colorado School of Medicine 12225, Aurora, Colorado, USA; 3 Our Lady of the Lake Regional Medical Center 23087, Baton Rouge, Louisiana, USA; 4 Santa Barbara Cottage Hospital 22854, Santa Barbara, California, USA; 5 Methodist University Hospital 23515, Memphis, Tennessee, USA; 6 UW Health SwedishAmerican Hospital 6938 , Rockford, Illinois, USA; 7 University of Illinois at Chicago, Rockford, Illinois, USA; 8 New York-Presbyterian Hospital 2034, New York, New York, USA; 9 The Brooklyn Hospital Center, Touro College of Pharmacy 471068 , New York, New York, USA; 10 College of Pharmacy, University of South Carolina 15525, Columbia, South Carolina, USA; 11 Philadelphia College of Pharmacy, Saint Joseph’s University 659977, Philadelphia, Pennsylvania, USA; 12 Cooper University Hospital 737464, Camden, New Jersey, USA; 13 University of Maryland School of Pharmacy, Baltimore, Maryland, USA; 14 Department of Pharmacy, Henry Ford Hospital 24016, Detroit, Michigan, USA; 15 Ascension St. Vincent Hospital 24104, Indianapolis, Indiana, USA; 16 Cape Fear Valley Health 3343, Fayetteville, North Carolina, USA; 17 University of Tennessee Health Science Center College of Pharmacy 15527, Memphis, Tennessee, USA; 18 University of Tennessee Medical Center 21823, Knoxville, Tennessee, USA; 19 St. Joseph’s/Candler Health System, Savannah, Georgia, USA; 20 Valley Hospital Medical Center 24899, Las Vegas, Nevada, USA; 21 Ochsner Clinic Foundation 633467 , New Orleans, Louisiana, USA; 22 Ochsner Clinical School, The University of Queensland 589787, New Orleans, Louisiana, USA; 23 Department of Pharmacy, Harper University Hospital, Detroit Medical Center 2956, Detroit, Michigan, USA; 24 Department of Medicine, Division of Infectious Diseases, School of Medicine, Wayne State University 2954, Detroit, Michigan, USA; JMI Laboratories, North Liberty, Iowa, USA

**Keywords:** eravacycline, multidrug-resistant, antimicrobial stewardship, immunocompromised

## Abstract

Infections from multidrug-resistant (MDR) bacteria lead to worse outcomes in immunocompromised patients. Eravacycline (ERV) is effective against MDR gram-negative and gram-positive bacteria, but its effects in immunocompromised populations remain unstudied. We aimed to evaluate clinical end points of immunocompromised patients receiving timely versus late ERV therapy. This multicenter, retrospective, observational study (October 2018 to September 2022) included adult immunocompromised patients hospitalized and treated with ERV for ≥72 h. The primary outcome was a composite of all-cause 30-day mortality, failure to improve clinically while on ERV, and/or microbial recurrence within 30 days of ERV initiation. Inverse probability of treatment weighting (IPTW) and Kaplan-Meier analyzes were used. Eighty-two patients from 17 US centers were included (median age 62 years; 59% male; intensive care unit admissions 57%). In the unadjusted cohort, timely ERV significantly reduced odds of clinical treatment failure (odds ratio [OR]: 0.321, 95% confidence interval [CI]: 0.129–0.800, *P* = 0.012) and microbial recurrence (OR: 0.545, 95% CI: 0.326–0.913, *P* = 0.034) compared to late ERV; in the IPTW-adjusted cohort, these associations remained significant with ORs of 0.675 (95% CI: 0.465–0.979, *P* = 0.029) for treatment failure and 0.384 (95% CI: 0.142–0.943, *P* = 0.041) for recurrence. Kaplan-Meier analysis showed a higher cumulative proportion of clinical treatment failure in the late ERV group (57%) compared to the timely ERV group (30%, *P* = 0.013), with a significantly longer time to clinical treatment failure in the timely ERV group (log-rank *P* = 0.034). Timely initiation of ERV in immunocompromised patients may improve clinical outcomes by reducing treatment failure and microbial recurrence compared to later initiation.

**IMPORTANCE:**

This multicenter, retrospective study evaluates the impact of timely versus late initiation of eravacycline (ERV) in immunocompromised patients with multidrug-resistant (MDR) bacterial infections. Immunocompromised patients face heightened risks of severe infections due to weakened immune defenses and limited treatment options, yet data on ERV use in this population are scarce. Our findings demonstrate that timely ERV initiation ≤72 h from index culture collection) significantly reduces clinical treatment failure and microbial recurrence compared to late initiation, with inverse probability of treatment weighting-adjusted odds ratios of 0.675 (*P* = 0.029) for treatment failure and 0.384 (*P* = 0.041) for recurrence. Kaplan-Meier analysis further confirms that delayed ERV therapy is associated with a higher cumulative incidence of clinical failure (57% vs 30%, *P* = 0.013). These results highlight the importance of early ERV initiation in optimizing outcomes for immunocompromised patients and addressing the growing challenge of MDR infections, emphasizing the need for further prospective studies to confirm these findings.

## INTRODUCTION

Multidrug-resistant (MDR) bacterial infections pose a significant global health threat, with pathogens like methicillin-resistant *Staphylococcus aureus* (MRSA), carbapenem-resistant *Enterobacteriaceae* (CRE), and vancomycin-resistant *Enterococcus* (VRE) demonstrating resistance to multiple antibiotics and complicating treatment (1). These pathogens resist treatment through mechanisms like drug efflux pumps, structural mutations, and enzyme production, diminishing antibiotic effectiveness (2). Although novel antibiotics such as ceftazidime-avibactam and meropenem-vaborbactam have been introduced, resistance has emerged rapidly in some clinical settings, and treatment options remain limited, emphasizing the urgent need for continued development of effective therapies against MDR infections (3, 4).

Immunocompromised individuals, including those with primary immunodeficiencies (PIDs) or those undergoing treatments for malignancies, transplants, or autoimmune diseases, are particularly vulnerable to MDR bacterial infections due to weakened immune defenses (5–7). Long-term immunosuppressive therapy, essential for conditions like hematological malignancies and organ transplants, further predisposes these patients to infection and adverse outcomes associated with antibiotic use (8, 9). Despite this high-risk profile, immunocompromised patients are often excluded from randomized controlled trials (RCTs) evaluating antibiotic safety and efficacy, leaving a gap in evidence-based guidance for managing infections in this population (10).

Bacterial infections in immunocompromised patients involve both gram-positive and gram-negative bacteria, with resistance exacerbating the challenge of effective treatment (11–13). Eravacycline (ERV), a novel fluorocycline with broad-spectrum activity against MDR gram-positive and gram-negative bacteria, including strains resistant to traditional tetracyclines, has shown promise for treating MDR infections (14–16). This study evaluates the timing of ERV administration to optimize its clinical outcomes, aiming to provide insights into effective use in this vulnerable population.

## MATERIALS AND METHODS

This was a multicenter, retrospective observational study of hospitalized adult patients conducted at 17 geographically distinct medical centers throughout the United States from October 2018 to September 2022. Patients meeting the following criteria were eligible for inclusion: (i) immunocompromised, (ii) ≥18 years of age, (iii) receipt of ERV for ≥72 continuous hours, and (iv) presence of an active bacterial infection, defined as microbiological evidence of pathogenic bacteria along with clinical symptoms, including fever (>38°C [100.4°F] or <36°C [96.8°F]), abnormal white blood cell count (WBC <4,000/µL or >12,000/µL or >10% bands), tachycardia (heart rate >90 bpm), or tachypnea (respiratory rate >20 breaths/min), while excluding colonization. Colonization was excluded based on the absence of systemic inflammatory signs (e.g., fever, leukocytosis, and tachycardia) and clinical judgment documented in the medical record. Patients were considered immunocompromised if they met one or more of the following criteria at the time of index hospitalization: receipt of cytotoxic chemotherapy, solid organ transplant (SOT), or bone marrow transplant (BMT) within the prior 90 days; diagnosis of HIV/AIDS with a CD4^+^ T cell count <200 cells/mm^3^; chronic corticosteroid use, defined as >40 mg of prednisone (or equivalent) daily for ≥30 days preceding ERV initiation; presence of an active hematologic malignancy (e.g., leukemia, lymphoma); history of splenectomy; absolute neutrophil count (ANC) < 500 cells/mm^3^ at time of ERV initiation, when not attributable to the conditions already listed above (e.g., secondary to drug toxicity, autoimmune disease, or idiopathic neutropenia).

Patients were excluded if any of the following occurred: transferred in from an outside hospital with known positive culture warranting ERV, pregnant women, prisoners, or any of the following within 72 h of index culture collection: (i) death, (ii) transferred to hospice, or (iii) transferred to an outside hospital. The primary outcome was a composite of clinical treatment failure defined as the presence of any of the following: (i) all-cause 30-day mortality, (ii) failure to improve clinically while on ERV, or (iii) microbial recurrence warranting treatment within 30 days of ERV initiation. Failure to improve clinically was defined as patients meeting one or more of the following criteria: (i) failure to resolve or improve infection-related abnormal WBC count; (ii) failure to resolve or improve abnormal temperature; and/or (iii) failure to improve signs or symptoms as documented by the treating physician, which resulted in a change in antibiotic therapy. Timely and late ERV therapy were defined as initiation of ERV within or after 72 h of index culture collection, respectively. The timing of ERV initiation was based on clinical judgment at each site and did not require confirmed culture or susceptibility results prior to administration. Combination therapy was defined as the concurrent use of at least one additional systemic antimicrobial agent with ERV for ≥48 h of overlapping administration.

For categorical data, Pearson chi-square or Fisher’s exact test was used as appropriate. For continuous data, the Wilcoxon rank-sum test was used. Kaplan-Meier analysis was performed to assess time to clinical treatment failure. Log-rank *P* value was reported to evaluate statistical significance between the timely and late ERV groups and the time to clinical treatment failure over the analysis period. To ensure groups were as similar as possible at the time of positive index culture collection and to allow unbiased comparisons between the timely and late ERV groups, analyzes were adjusted for possible confounding with IPTW using baseline covariates with a *P* ≤ 0.1 present in at least 10% of the total population (17). Covariate balance was assessed through the Kolmogorov-Smirnov goodness-of-fit statistic and standardized mean difference, with >0.1 and >0.2 indicating an imbalance, respectively. The predictive ability of the propensity score model was evaluated by the area under the receiver operating characteristic curve. Bivariate regression analyzes were subsequently conducted to compare primary and secondary outcomes between the weighted timely and late ERV cohorts. Odds ratio (OR) and adjusted OR with 95% confidence interval (CI) were calculated. Statistical analyzes were performed using IBM SPSS Statistics, version 29.0 (IBM Corp., Armonk, NY, USA).

## RESULTS

### Baseline demographics and clinical characteristics

This study evaluated 82 immunocompromised patients receiving timely ERV (*n* = 40) or late (*n* = 42) ERV. Baseline demographic and clinical characteristic data between groups are listed in Table 1. Patient cohorts were well balanced at baseline; however, significantly fewer patients in the timely ERV group received active antibiotic therapy prior to ERV initiation (28% vs 69%; *P* < 0.001) and concomitant intravenous (IV) antibiotic therapy (25% vs 52%; *P* = 0.011) compared to the late ERV group, respectively.

**TABLE 1:** Baseline and clinical characteristics^*b*^

Characteristics^*a*^	Timely ERV (*n* = 40)	Late ERV (*n* = 42)	*P* value
Age (years)	61 (51–70)	62 (52–69)	0.989
Male	23 (57.5)	25 (59.5)	0.515
Race			
African American	10 (25)	7 (16.7)	0.172
Caucasian	25 (62.5)	31 (73.8)	0.194
Hispanic	4 (10)	3 (7.1)	0.643
Other	1 (2.5)	1 (2.4)	0.512
Baseline SCr (mg/dL)	0.86 (0.59–1.24)	0.89 (0.62–1.38)	0.810
Admitted from			
Home	26 (65)	27 (64.3)	0.504
NH/LTC	6 (15)	6 (14.3)	0.553
Inpatient rehab	2 (5)	1 (2.4)	0.472
Referral from clinic	1 (2.5)	1 (2.4)	0.734
Transfer from outside hospital	5 (12.5)	7 (16.7)	0.431
Charlson comorbidity index	4 (2–6)	5 (3–7)	0.375
Immunocompromising condition			
Bone marrow transplant	6 (15)	2 (4.8)	0.234
Chronic steroid use	6 (15)	9 (21.4)	0.641
Cytotoxic chemotherapy	18 (45)	12 (28.6)	0.189
HIV/AIDS with CD4 <200	1 (2.5)	2 (4.8)	>0.999
Leukemia or lymphoma	10 (25)	8 (19)	0.701
Neutropenia	5 (12.5)	8 (19)	0.611
Splenectomy	1 (2.5)	3 (7.1)	0.644
Solid organ transplant	7 (17.5)	3 (7.1)	0.273
Patients with ≥2 conditions	14 (35)	17 (40.5)	0.777
SOFA score	3 (0–4)	4 (1–7)	0.156
APACHE II score	25 (19–28)	24 (19–28)	0.403
ICU admission	22 (55)	25 (60)	0.679
In ICU at index culture collection	8 (20)	14 (33)	0.173
Culture specimen			
Blood	11 (28)	12 (29)	0.914
Fluid	10 (25)	12 (29)	0.715
Respiratory	12 (30)	10 (24)	0.527
Tissue	7 (18)	8 (19)	0.856
ID consult	39 (98)	41 (98)	0.134
Active antibiotic before ERV	11 (28)	29 (69)	<0.001
Amikacin	0 (0)	1 (2.4)	>0.999
Ceftazidime/avibactam	1 (2.5)	3 (7.1)	0.644
Ertapenem	0 (0)	4 (9.5)	0.137
Gentamicin	0 (0)	2 (4.8)	0.496
Meropenem	3 (7.5)	5 (11.9)	0.764
Meropenem/vaborbactam	0 (0)	2 (4.8)	0.496
Tigecycline	0 (0)	1 (2.4)	>0.999
Vancomycin	7 (17.5)	11 (26.2)	0.494
Reason for transition to ERV			
Consolidation of therapy	5 (12.5)	17 (40.5)	0.009
Safety/tolerability	3 (7.5)	8 (19)	0.226
Carbapenem-sparing	3 (7.5)	4 (9.5)	>0.999
Concomitant IV antibiotic	10 (25)	22 (52)	0.021
Amikacin	2 (5)	4 (9.5)	0.717
Ciprofloxacin	1 (2.5)	1 (2.4)	>0.999
Colistin	1 (2.5)	2 (4.8)	>0.999
Gentamicin	0 (0)	2 (4.8)	0.496
Imipenem	1 (2.5)	0 (0)	0.981
Levofloxacin	0 (0)	2 (4.8)	0.496
Meropenem	2 (5)	5 (11.9)	0.471
Tobramycin	1 (2.5)	4 (9.5)	0.386
Trimethoprim/sulfamethoxazole	1 (2.5)	2 (4.8)	>0.999
Duration of antibiotic therapy	7 (4.4–10.9)	8.7 (6.2–16)	0.439

^*a*^ Data are presented as “number (%)” or “median (interquartile range),” as appropriate. Timely and late ERV are defined as receipt of ERV within or after 72 h of index culture collection, respectively.

^*b*^ APACHE II, acute physiology and chronic health evaluation; ERV, eravacycline; ICU, intensive care unit; ID, infectious diseases; IV, intravenous; NH/LTC, nursing home/long-term care; SCr, serum creatinine; SOFA, sequential organ failure assessment.

**Fig 1:**
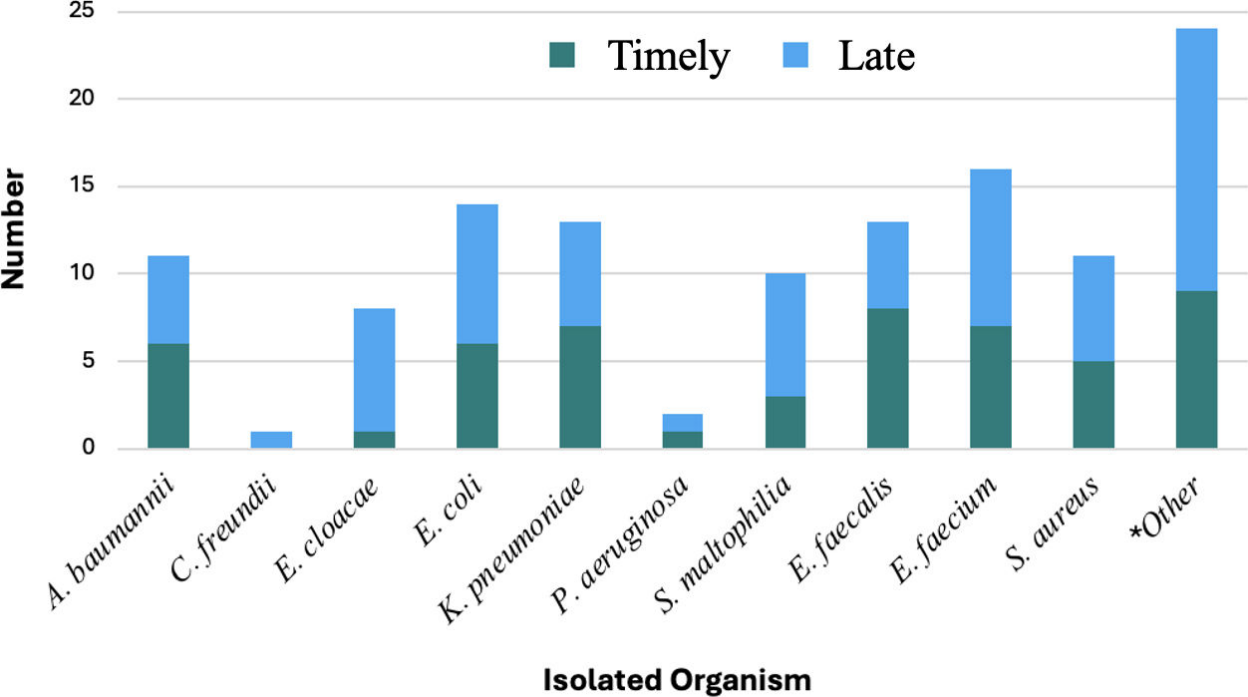
Index cultured organisms treated with eravacycline. *Other: *Citrobacter koseri*, *Klebsiella oxytoca*, *Morganella morganii*, *Proteus mirabilis*, *Serratia marcescens*, and *Streptococcus* spp.

Median (interquartile range [IQR]) age was 62 (53–70) years and 59% were male. Hospital length of stay was 28 (13–42) days and 67% were admitted to the intensive care unit (ICU). SOFA and APACHE II scores were 4 (1–7) and 16 (11–20), respectively. Most patients had *Enterobacterales* (59%) or *Enterococci* (37%) spp. infections (Fig. 1). Of those, 21% were carbapenem-resistant *Enterobacterales* (CRE) and 19% were vancomycin-resistant *Enterococci* (VRE), respectively. Infectious disease consultation was obtained in 98% of patients.

The propensity score distribution between patients receiving timely versus late ERV was adequately balanced after IPTW, as demonstrated by the Kolmogorov-Smirnov test with pre- and post-IPTW *P* values of 0.022 and 0.469, respectively. The prediction ability of the propensity score model with an area under the receiver operating characteristic curve was 80%. Unadjusted and IPTW-adjusted primary and secondary end points are presented in Table 2.

**TABLE 2:** Clinical end points^*b*^

Outcomes^*a*^	Unadjusted cohort IPTW cohort				IPTW cohort	
	Timely ERV (*n* = 40)	Late ERV (*n* = 42)	OR (95% CI)	*P* value	aOR (95% CI)	*P* value
Clinical treatment failure	12 (30)	24 (57)	0.321 (0.129–0.800)	0.012	0.675 (0.465–0.979)	0.029
All-cause 30-day mortality	12 (30)	14 (33)	0.857 (0.337–2.177)	0.466	0.912 (0.135–1.263)	0.653
Worsen/fail to improve clinically	0 (0)	3 (7)	1.097 (0.367–3.276)	0.544	1.444 (0.446–4.678)	0.750
Microbial recurrence	0 (0)	7 (17)	0.545 (0.326–0.913)	0.034	0.384 (0.142–0.943)	0.041
Hospital length of stay (days)	20 (11–36)	31 (15–46)	0.995 (0.982–1.008)	0.088	0.755 (0.368–1.549)	0.194
ICU length of stay (days)	10 (4–22)	23 (13–50)	0.996 (0.970–1.022)	0.108	0.776 (0.059–2.190)	0.374
60-day hospital readmission	19 (48)	17 (40)	0.417 (0.495–4.054)	0.056	0.384 (0.142–1.043)	0.071

^*a*^ Data are presented as “number (%)” or “Odds ratio (95% confidence interval),” as appropriate. Timely and late ERV are defined as receipt of ERV within or after 72 h of index culture collection, respectively.

^*b*^ aOR, adjusted odds ratio; ERV, eravacycline; ICU, intensive care unit; IPTW, inverse probability of treatment weighting; OR, odds ratio.

### Primary and secondary outcomes

In the unadjusted cohort, timely ERV was associated with significantly lower odds of clinical treatment failure (OR: 0.321, 95% CI: 0.129–0.800, *P* = 0.012) and microbial recurrence (OR: 0.545, 95% CI: 0.326–0.913, *P* = 0.034) compared to late ERV. In the IPTW cohort, these associations remained significant with adjusted ORs of 0.675 (95% CI: 0.465–0.979, *P* = 0.029) for clinical treatment failure and 0.384 (95% CI: 0.142–0.943, *P* = 0.041) for microbial recurrence. No significant differences were observed in all-cause 30-day mortality, clinical worsening, hospital length of stay, ICU length of stay, or 60-day hospital readmission between the timely and late ERV groups in either cohort.

**Fig 2:**
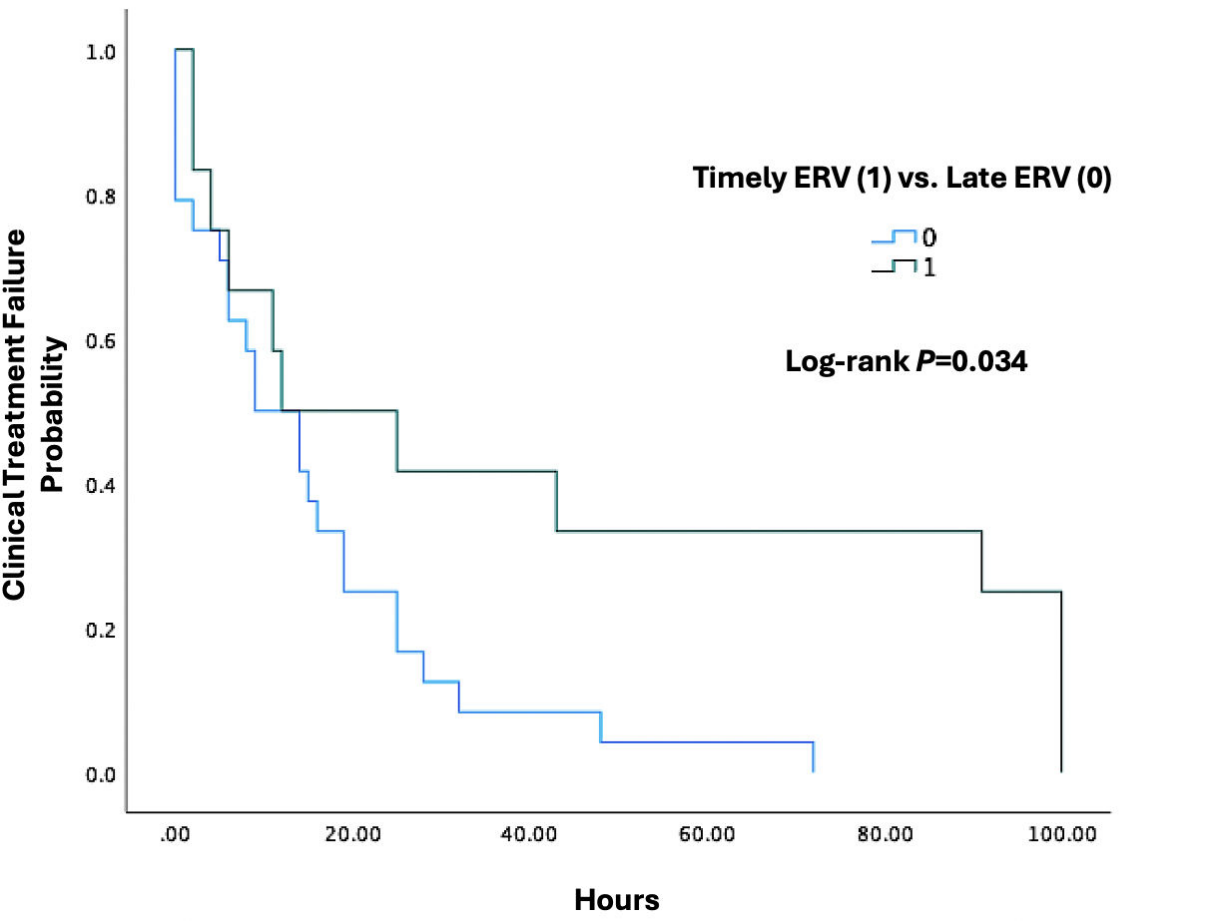
Kaplan-Meier curve for time to clinical treatment Failure. This Kaplan-Meier curve illustrates the probability of clinical treatment failure over time (measured in hours) in two groups: patients who received timely eravacycline (ERV) therapy (ERV = 1, green line) and those who received it later (ERV = 0, blue line). The *y*-axis represents the cumulative probability of clinical treatment failure, while the *x*-axis represents time in hours. Patients in the timely ERV group demonstrated a lower cumulative probability of clinical treatment failure compared to those in the late group. A statistically significant difference between the two groups was observed, as indicated by the log-rank test (*P* = 0.034). The median time to clinical treatment failure was 27 h (interquartile range [IQR], 15–60 hours) in the timely group versus 13 h (IQR, 6–20 h) in the late group, highlighting the association between earlier ERV initiation and delayed or reduced likelihood of treatment failure. Censored patients include those who had not yet experienced treatment failure based on the definition of the composite outcome.

Kaplan-Meier analysis (Fig. 2) demonstrated a significant increase in the cumulative proportion of patients meeting the primary end point of clinical treatment failure (12/40 [30%] for the timely ERV group and 24/42 [57%] of the late ERV group; *P* = 0.013). Furthermore, the time to clinical treatment failure was significantly longer in the timely ERV group compared to the late ERV group (log-rank *P* = 0.034). The median time to clinical treatment failure was 27 h (IQR, 15–60 h) in the timely group versus 13 h (IQR, 6–20 h) in the late group, further supporting the benefit of earlier ERV initiation in this population.

## DISCUSSION

This study aimed to evaluate the clinical outcomes of immunocompromised patients receiving ERV therapy, offering insights into its efficacy and tolerability in this vulnerable population. The main takeaway is that timely initiation of ERV (within 72 h of index culture collection) in immunocompromised patients with bacterial infections significantly reduced clinical treatment failure and microbial recurrence compared to delayed initiation. This finding was consistent in unadjusted and IPTW-adjusted analyses and underscores the critical importance of prompt antimicrobial therapy in improving clinical outcomes for this vulnerable patient population.

Immunocompromised patients are at an increased risk of severe bacterial infections (18–20). This vulnerability extends beyond those with hematologic malignancies and neutropenia to include patients with SOTs, BMTs, chronic corticosteroid use, HIV/AIDS with CD4 <200 cells/mm^3^, splenectomy, and other forms of immunosuppression such as recent cytotoxic chemotherapy or the presence of multiple immunocompromising conditions (5–7). These diverse underlying states contribute to impaired host defenses, increasing the risk of both initial infection and poor clinical outcomes. Notably, in our cohort, a significant proportion of patients had more than one immunocompromising condition, further compounding their susceptibility to complications from MDR bacterial infections.

Historically, gram-positive bacteria have been the predominant pathogens in immunocompromised patients; however, recent data indicate a shift, with ggram-negative bacteria re-emerging as frequent pathogens (12, 13). Many of these pathogens have developed resistance to commonly used antimicrobial agents, highlighting the urgent need for novel therapeutic options. Higher antibiotic utilization in this population contributes to increased rates of antibiotic resistance and a heightened risk of *Clostridioides difficile* infections, further complicating their clinical management and adding to the healthcare burden, including increased hospitalization costs (21).

ERV, with its broad-spectrum activity against MDR organisms like VRE and CRE (14, 22, 23), offers a potential solution in this high-risk group. However, its use in immunocompromised patients has been largely empirical, due to delays in incorporating ERV into automated susceptibility panels across institutions during much of the study period. As such, many patients in this cohort received ERV prior to organism identification or susceptibility confirmation. Among those with documented pathogens, recurrence was due to the same organism identified in the index culture. Unfortunately, repeat susceptibility testing was inconsistently performed, limiting our ability to evaluate resistance development at the time of recurrence. This gap highlights the need for more robust microbiologic follow-up in future studies.

It is important to note that ERV and other novel agents are rarely tested in high-risk populations, such as immunocompromised patients. RCT data are often limited for these patients because of their heightened susceptibility to adverse events and the complexity of their medical conditions, which can complicate trial enrollment and protocol adherence (24–26). Although prospective and randomized trials are necessary to establish the efficacy and safety of ERV definitively, retrospective health outcomes studies are essential in the interim to provide valuable insights into the potential effectiveness of ERV in real-world settings for this population. While this study offers preliminary evidence of the potential of ERV, further rigorous retrospective and prospective research is needed to confirm these findings and determine the optimal therapeutic applications of ERV in immunocompromised patients.

The current study does have implications for clinical practice, particularly in settings where timely initiation of effective antimicrobial therapy is critical. While ERV shows promise as an option for empirical therapy in high-risk neutropenic febrile patients, intra-abdominal infections in non-neutropenic immunocomromised individuals, and as step-down therapy for resistant infections, further research is necessary to support its widespread use. In our study, ERV was used as monotherapy for most patients; however, combination therapy may have been employed during the initial 48 h in certain cases. Potential reasons include concern for incomplete pathogen coverage prior to susceptibility results, clinical instability at the time of ERV initiation, polymicrobial infections involving organisms not reliably covered by ERV (e.g., *Pseudomonas aeruginosa*), and general caution due to limited real-world experience with ERV monotherapy in immunocompromised populations. Although we did not collect specific documentation on the rationale for combination therapy, these considerations reflect common clinical decision-making and underscore the need for further data to guide optimal use of ERV in this setting. The long half-life and pharmacokinetics of ERV suggest it could also be suitable for outpatient therapy, particularly as a step-down treatment for clinically stable patients (27, 28).

This research has study design limitations. The retrospective nature introduces potential biases, such as selection bias and information bias, which can affect the validity of the results. The study’s multicenter approach, while enhancing generalizability, may also introduce variability in treatment practices. The relatively small sample size may limit the statistical power and applicability to all immunocompromised subpopulations. For example, while a greater proportion of patients in the timely ERV group received monotherapy compared to those in the late group (as shown in Table 1), we did not collect detailed site-level data to assess the rationale behind monotherapy versus combination therapy decisions. This limits our ability to draw conclusions about the clinical factors driving treatment selection. Additionally, not all potential confounding factors were accounted for, despite using IPTW. Finally, the observational design precludes establishing a causal relationship between timely ERV initiation and improved outcomes. Future prospective studies are warranted to confirm these findings and further explore the benefits of prompt ERV therapy in larger and more diverse cohorts of immunocompromised patients. These studies should aim to standardize treatment protocols and control for additional confounding factors to provide more robust evidence.

In conclusion, while timely initiation of ERV appears to be a promising strategy for reducing clinical treatment failure and microbial recurrence in immunocompromised patients with bacterial infections, additional prospective research is needed to validate these findings and optimize treatment protocols for this high-risk group. Integrating ERV into clinical practice could improve patient outcomes and offer a critical tool in managing MDR bacterial infections, ultimately addressing a significant clinical and financial burden in healthcare systems.
